# Hierarchical fixed points of strictly pseudo contractive mappings for variational inequality problems

**DOI:** 10.1186/2193-1801-2-540

**Published:** 2013-10-17

**Authors:** Tanom Chamnarnpan, Nopparat Wairojjana, Poom Kumam

**Affiliations:** Department of Mathematics, Faculty of Science, King Mongkut’s University of Technology Thonburi (KMUTT), 126 Pracha Uthit Road, Bang Mod, Thrung Khru, Bangkok, 10140 Thailand; Department of Mathematics and Statistics, Faculty of Science, Valaya Alongkorn Rajabhat University under the Royal Patronage, Pahonyotin Road, Pathumtani, 13180 Thailand

**Keywords:** Strong convergence, Strictly pseudo-contractive mapping, Hilbert spaces, Variational inequality, Hierarchical fixed point problem

## Abstract

In this paper, we introduce a new iterative scheme that converges strongly to a common fixed point of a countable family of strictly pseudo-contractive mappings in a real Hilbert space which is also a solution of variational inequality problem related to quadratic minimization problems. Our results extend ones of Yao et al. [Math. and Comput. Modell. 52(9-10):1697–1705, 2010], Gu et al. [J. Appl. Math. 2011:17 p., 2011] and some authors.

## Introduction

Throughout this paper, we assume that *H* is a real Hilbert space with inner product and norm denoted by 〈·,·〉 and ∥·∥, respectively and let *C* is a nonempty closed and convex subset of *H*. A mapping *f*:*C*→*H* is called a *contraction* on *C* if there exists a constant *ρ* ∈ [ 0,1) such that

A mapping *T*:*C*→*H* is called a *nonexpansive* on *C* if

A mapping *M*:*H*→*H* is called a *strongly monotone operator* with coefficient *α* if there exists a constant *α* > 0 such that

and *M* is called a *monotoneoperator* if

It is well known that the mapping (*I*−*T*) is a monotone operator, if *T* is a nonexpansive mapping and *I* is a identity mapping.

A mapping *T*:*C*→*H* is called *k*-*strictpseudo*-*contraction* if there exists a constant *k* ∈ [ 0,1) such that

If there exists a point *x* ∈ *C* such that *x* = *Tx*, then *x* is said to be fixed point of *T*. We denote the set of all fixed pionts of *T* by *F*(*T*). It is well known that *F*(*T*) is closed and convex if *T* is nonexpansive.

Note that the class of *k*-*strictpseudo*-*contraction* mappings includes the class of nonexpansive mappings on *C* as a subclass. i.e., *T* is nonexpansive if and only if *T* is 0-*strictpseudo*-*contraction*. Recently, many authors have been devoting the studies on the problems of finding fixed points for *k*-*strictpseudo*-*contraction* mappings; see Acedo and Xu (2007); Cho et al. (2009); Jung (2010); Jung (2011); Zhou (2008) and the references therein.

A variational inequality in a real Hilbert space *H* is formulated as finding a point *x*^∗^ ∈ *C* such that1

where *F*:*C*→*H* is a nonlinear mapping. We denote the set of solution of (1) by *VI*(*C*,*F*). If *F* is a monotone operator, then (1) is also known as *a monotone variational inequality*.

For given nonlinear operators *F*,*g*, we consider the problem of finding *u* ∈ *H* such that2

which is known as *the general variational inequality problem*. For given nonlinear operators *F*,*g*,*h*, we consider the problem of finding *u* ∈ *H* : *h*(*u*) ∈ *C* such that3

which is called *the extended general variational inequality*.

The variational inequalities have been studied widely and are being used as a mathematical programming tool in modeling a wide class of problems arising in several branches of pure and applied sciences; see Baiocchi and Capelo (1984); Giannessi and Maugeri (1995); Kinderlehrer and Stampacchia (1980). For general variational inequalities and extended general variational inequalities, we can refer Noor (2004,2009); Noor et al. ([Bibr CR18][Bibr CR19]) and references therein.

It is well-known that the variational inequality (1) is equivalent to the fixed point equation4

where *γ* > 0 and *P*_*C*_ is the metric projection of *H* onto *C* which assigns, to each *x* ∈ *H*, the unique point in *C*, denoted *P*_*C*_[ *x*], such that

Therefore, fixed point algorithms can be applied to solve variational inequalities.

The following problem is called a *hierarchical fixed point problem*: Find *x*^∗^∈*F*(*T*) such that5

where *S*:*C*→*H* be a mapping. It is known that the hierarchical fixed point problem (5) links with some monotone variational inequalities and convex programming problems; see Gu et al. (2011); Yao et al. (2010).

In order to solve the hierarchical fixed point problem (5), Moudafi (2007) intoduced the following Krasnoselski-Mann algorithm:6

where *S*,*T*:*C*→*C* are two nonexpansive mappings, {*α*_*n*_} and {*β*_*n*_} are two sequences in (0,1). Then he showed that {*x*_*n*_} converges weakly to a fixed point of *T* which is a solution of problem (5). For obtaining a strong convergence result, in Mainge and Moudafi (2007) and Marino and Xu (2011) introduced the following algorithm:7

where *f*:*C*→*C* is a contraction mapping, *S* and *T*:*C*→*C* are two nonexpansive mappings, {*α*_*n*_} and {*β*_*n*_} are two sequences in (0,1). Then they showed that {*x*_*n*_} converges strongly to a fixed point of *T* which is a solution of problem (5).

On the other hand, Cianciaruso et al. (2009) introduced a two step algorithm to solve the problem (5) as follows:8

where *f*:*C*→*C* is a contraction mapping, *S* and *T*:*C*→*C* are two nonexpansive mappings, {*α*_*n*_} and {*β*_*n*_} are two sequences in (0,1). Under some certain restrictions on parameters, the authors proved the sequence {*x*_*n*_} generated by (8) converges strongly to *x*^∗^∈*F*(*T*), which is a unique solution of the following variational inequality:9

By changing the restrictions on parameters, the authors obtained another result on the iterative scheme (8), i.e., the sequence {*x*_*n*_} generated by (8) converges strongly to *x*^∗^∈*F*(*T*), which is a unique solution of the following variational inequality:10

where *τ* ∈ (0,*∞*) is a constant.

In 2010, Yao et al. (2010) modified the two step algorithm (8) to extend Range of *f* from *C* to *H* by using the metric projection of *H* onto *C*. They introduced the following iterative scheme:11

where *f*:*C*→*H* is a contraction mapping, *S* and *T*:*C*→*C* are two nonexpansive mappings, {*α*_*n*_} and {*β*_*n*_} are two sequences in (0,1). The authors proved the sequence {*x*_*n*_} generated by (11) converges strongly to *x*^∗^ ∈ *F*(*T*), which is a unique solution of one of the variational inequalities (9) and (10).

In 2011, Gu et al. (2011) introduced the following iterative algorithm:12

where *f*:*C*→*H* is a contraction mapping, *S*:*C*→*H* is a nonexpansive mapping,  is a countable family of nonexpansive mappings, *α*_0_=1, {*α*_*n*_} and {*β*_*n*_} are two sequences in (0,1). The authors proved the sequence {*x*_*n*_} generated by (12) converges strongly to *x*^∗^ ∈ *F*(*T*), which is a unique solution of one of the variational inequalities (9) and (10).

In this paper, motivated and inspired by the results of Gu et al. (2011), we introduce and study the following iterative scheme:13

where *V*_*i*_=*k*_*i*_*I*+(1−*k*_*i*_)*T*_*i*_ and  is a countable family of *k*_*i*_-strict pseudo-contraction mappings. Under some certain condition on parameters, we first prove that the sequence {*x*_*n*_} generated by (13) converges strongly to  which is a unique solution of the following variational inequality:14

By changing the restrictions on parameters, we also prove that the sequence {*x*_*n*_} generated by (13) converges strongly to , which is a unique solution of the following variational inequality:15

where *τ* ∈ (0,*∞*) is a constant. It is easy to see that, if *k*_*i*_=0 for each *i*≥1, then our algorithm (13) is reduced to algorithm (12) of Gu et al. Also our results extend the corresponding one of Yao et al. (2010); Xu (2004); Cianciaruso et al. (2009); Moudafi (2000) and Gu et al. (2011) from the countable family of nonexpansive mappings to more general the countable family of strictly pseudo contraction mappings.

## Preliminaries

This section collects some lemma which be use in the proofs for the main results in the next section. Some of them are known; others are not hard to derive.

We will use the following notation:

(i) → for strong convergence and ⇀ for weak convergence.
(ii)denotes the weak *ω*-limit set of {*x*_*n*_}.

### Lemma 1

Browder (1976) Let *H* be a Hilbert space, *C* is a closed convex subset of *H* and *T*:*C*→*C* be a nonexpansive mapping with *F*(*T*)≠*∅*. If {*x*_*n*_} is a sequence in *C* weakly converging to *x* and if {(*I*−*T*)*x*_*n*_} converges strongly to *y*, then (*I*−*T*)*x*=*y*; in particular, if *y*=0 then *x* ∈ *F*(*T*).

### Lemma 2

Acedo and Xu (2007) Let *C* be a nonempty closed convex subset of a real Hilbert space *H*. If *T*:*C*→*C* is a *k*-strict pseudo-contraction, then the mapping *I*−*T* is demiclosed at 0. That is, if {*x*_*n*_} is a sequence in *C* weakly converging to *x* and {(*I*−*T*)*x*_*n*_} converges strongly to 0, then (*I*−*T*)*x*=0.

### Lemma 3

Let *x* ∈ *H* and *z* ∈ *C* be any points. Then we have the following:

1. That *z*=*P*_*C*_[ *x*] if and only if there holds the relation:
2. That *z*=*P*_*C*_[ *x*] if and only if there holds the relation:
3. There holds the relation:

Consequently, *P*_*C*_ is nonexpansive and monotone.

### Lemma 4

Marino and Xu (2006) Let *H* be a Hilbert space, *C* be a closed convex subset of *H*, *f*:*C*→*H* be a contraction with coefficient 0 < *ρ* < 1 and *T* : *C* → *C* be a nonexpansive mapping. Then, for 0 < *γ* < *γ̄*/*ρ*, for *x*,*y*∈*C*,

1. the mapping (*I*−*f*) is strongly monotone with coefficient (1−*ρ*) that is
2. the mapping (*I*−*T*) is monotone, that is

### Lemma 5

Xu (2002) Assume that {*a*_*n*_} is a sequence of nonnegative numbers such that

where {*γ*_*n*_} is a sequence in (0,1) and {*δ*_*n*_} is a sequence in  such that

,

or .

Then lim_*n*→*∞*_*a*_*n*_=0.

### Lemma 6

Acedo and Xu (2007) Let *C* be a closed convex subset of *H*. Let {*x*_*n*_} be a bounded sequence in *H*. Assume that

1. The weak *ω*-limit set *ω*_*w*_(*x*_*n*_)⊂*C*,
2. For each *z* ∈ *C*, lim_*n*→*∞*_∥*x*_*n*_−*z*∥ exists.

Then {*x*_*n*_} is weakly convergent to a point in *C*.

### Lemma 7

Zhou (2008) Let *H* be a real Hilbert space, *C* be a closed and convex subset of H, and *T* be a *k*-strict pseudo-contraction mapping on *C*, then *F*(*T*) is closed convex, so that the projection *P*_*F*(*T*)_ is well defined.

### Lemma 8

Zhou (2008) Let *H* be a Hilbert space, *C* be a closed and convex subset of *H*, and *T*:*C*→*H* be a *k*-strict pseudo-contraction mapping. Define a mapping *V*:*C*→*H* by *Vx*=*λx*+(1−*λ*)*Tx* for all *x* ∈ *C*. Then, as *λ* ∈ [*k*,1), *V* is a nonexpansive mapping such that *F*(*V*) = *F*(*T*).

### Lemma 9

Gu et al. (2011) Let *H* be a Hilbert space and *C* be a nonempty closed and convex subset of *H*. Let *T* be a nonexpansive mapping of *C* into itself such that *F*(*T*)≠*∅*. Then ∥*Tx*−*x*∥^2^≤2〈*x*−*Tx*,*x*−*x*^′^〉, ∀*x*^′^ ∈ *F*(*T*),∀*x* ∈ *C*.

## Main results

Let us consider the net iterative scheme as follows:16

where *V*_*i*_=*k*_*i*_*I*+(1−*k*_*i*_)*T*_*i*_, *f*:*C*→*H* is a *ρ*-contraction mapping, *S*:*C*→*H* is a nonexpansive mapping,  is a countable family of *k*_*i*_-strict pseudo-contraction mappings and . Set *α*_0_=1, {*α*_*n*_}⊂(0,1) is a strictly decreasing sequence and {*β*_*n*_}⊂(0,1). As we will see the convergence of the scheme depends on the choice of the parameters {*α*_*n*_} and {*β*_*n*_}. We list some possible hypotheses on them:

- ● (H1) there exists *γ* > 0 such that *β*_*n*_≤*γα*_*n*_;
- ● (H2) lim_*n*→*∞*_*β*_*n*_/*α*_*n*_=*τ* ∈ [ 0,*∞*);
- ● (H3) lim_*n*→*∞*_*α*_*n*_=0 and ;
- ● (H4) ;
- ● (H5) ;
- ● (H6) lim_*n*→*∞*_|*α*_*n*_−*α*_*n*−1_|/*α*_*n*_=0;
- ● (H7) lim_*n*→*∞*_|*β*_*n*_−*β*_*n*−1_|/*β*_*n*_=0;
- ● (H8) lim_*n*→*∞*_[ |*α*_*n*_−*α*_*n*−1_|+|*β*_*n*_−*β*_*n*−1_|]/*α*_*n*_*β*_*n*_=0;
- ● (H9) there exists a constant *K* > 0 such that● .

### Proposition 1

Assume that (H1) holds. Then {*x*_*n*_} and {*y*_*n*_} are bounded.

### Proof

Let  Then we have17

So, by induction, one can obtain that18

Hence {*x*_*n*_} is bounded. Of course {*y*_*n*_} is bounded too. □

### Proposition 2

Suppose that (H1) and (H3) hold. Also, assume that either (H4) and (H5) hold, or (H6) and (H7) hold. Then

(i) {*x*_*n*_} is asymptotically regular, that is,19
(ii) the weak cluster points set .

### Proof

Set . From (16) and since *P*_*C*_ is a nonexpansive mapping, we have2021

By definition of *y*_*n*_ one obtain that22

So, substituting (22) in (21), we obtain23

By Proposition 1, we say

So, we have24

So, if (H4) and (H5) hold, we obtain the asymptotic regularity by Lemma 5, if instead, (H6) and (H7) hold, from (H1), we can write25

By Lemma 5, we obtain the asymptotics regularity.

In order to prove (2), since *V*_*i*_*x*_*n*_ ∈ *C* for each *i* ≥ 1 and , we have26

Now, fixing , from (16), we have

It follows that 27

Now, from Lemma 9 and (27), we get 

By (H1) and (H3), it follows that *β*_*n*_→0, as *n*→*∞*, so that28

Since  for each *i*≥1 and {*α*_*n*_} is strictly decreasing, one has29

Hence, we obtain

Since {*x*_*n*_} is asymptotically regular and demiclosedness principle, we obtain the proposition. □

### Corollary 1

Suppose that the hypotheses of Proposition 2 hold. Then

(i) lim_*n*→*∞*_∥*x*_*n*_−*y*_*n*_∥=0;
(ii) lim_*n*→*∞*_∥*x*_*n*_−*V*_*i*_*y*_*n*_∥=0, ∀*i*≥1;
(iii) lim_*n*→*∞*_∥*y*_*n*_−*V*_*i*_*y*_*n*_∥=0, ∀*i*≥1.

### Proof

To prove (*i*), we can observe that

Since *β*_*n*_→0 as *n*→*∞*, we obtain (*i*).To prove (*ii*), we observe that

and

Since ∥*y*_*n*_−*x*_*n*_∥→0 and ∥*x*_*n*_−*V*_*i*_*x*_*n*_∥→0 as *n*→*∞*, ∀*i*≥1, then ∥*y*_*n*_−*V*_*i*_*x*_*n*_∥→0, that is, we obtain (*ii*). To prove (*iii*), we can observe that

By (*i*) and (*ii*), we obtain (*iii*). □

### Theorem 1

Let *C* be a nonempty closed and convex subset of a real Hilbert space *H*. Let *f*:*C*→*H* be a *ρ*-contraction mapping, *S*:*C*→*H* be a nonexpansive mapping and  be a countable family of *k*_*i*_-strict pseudo-contraction mappings and . Let *α*_0_=1, and *x*_1_ ∈ *C* and define the sequence {*x*_*n*_} by30

where {*α*_*n*_}⊂(0,1) and {*α*_*n*_} is a strictly decreasing sequence, *V*_*i*_=*k*_*i*_*I*+(1−*k*_*i*_)*T*_*i*_, {*β*_*n*_}⊂(0,1) and {*α*_*n*_} and {*β*_*n*_} are sequences satisfying the conditions (H2) with *τ*=0, (H3), either (H4) and (H5), or (H6) and (H7). Then the sequence {*x*_*n*_} converges strongly to a point , which is the unique solution of the variational inequality:31

### Proof

First of all, since  is a contraction. By Banach contraction principle, so there exists a unique  such that , Moreover, from Lemma 3(1), we have

Since (H2) implies (H1), thus {*x*_*n*_} is bounded. Moreover, since either (H4) and (H5) or (H6) and (H7) then {*x*_*n*_} is asymptotically regular. Similarly, by Proposition 2, the weak cluster points set of *x*_*n*_, that is, *ω*_*w*_(*x*_*n*_), is a subset of .

Let  be a subsequence of {*x*_*n*_} such that

and ’. So, it follows that . Then, we also have

Set , we obtain32

By Lemma 3(1), we have33

From (32) and (33), it follows that

∥*x*_*n*+1_ − *z*∥^2^

Let  and  for all *n*≥1. Since

 and , we have

Hence, by Lemma 5, we conclude that *x*_*n*_→*z* as *n*→*∞*. □

### Remark 1

In the iterative scheme (30), if we set *f*≡0, then we get . In this case, from (31), it follows that

That is

Therefore, the point *z* is the unique solution to the following quadratic minimization problem:

By changing the restrictions on parameters in Theorem 1, we obtain the following results.

### Theorem 2

Let *C* be a nonempty closed and convex subset of a real Hilbert space *H*. Let *f*:*C*→*H* be a *ρ*-contraction mapping, *S*:*C*→*C* be a nonexpansive mapping and  be a countable family of *k*_*i*_-strict pseudo-contraction mappings and . Let *α*_0_ = 1, and *x*_1_ ∈ *C* and define the sequence {*x*_*n*_} by34

where {*α*_*n*_}⊂(0,1) and {*α*_*n*_} is a strictly decreasing sequence, *V*_*i*_=*k*_*i*_*I*+(1−*k*_*i*_)*T*_*i*_, {*β*_*n*_}⊂(0,1) and {*α*_*n*_} and {*β*_*n*_} are sequences satisfying the conditions (H2) with *τ* ∈ (0,*∞*), (H3), (H8) and (H9). Then the sequence {*x*_*n*_} converges strongly to a point , which is the unique solution of the variational inequality:35

### Proof

First, we shows that (49) has the unique solution. Let *x*^′^ and *x*^∗^ be two solutions. Then, since *x*^′^ is solution, for *y*=*x*^∗^ one has36

and37

Adding (36) and (37), we obtain

so *x*^′^=*x*^∗^. Also now the condition (H2) with 0<*τ*<*∞* implies (H1) so the sequence {*x*_*n*_} is bounded. Moreover, since (H8) implies (H6) and (H7), then {*x*_*n*_} is asymptotically regular. Similarly, by Proposition 2, the weak cluster points set of *x*_*n*_, i.e., *ω*_*w*_(*x*_*n*_), is a subset of .

From (20)-(24), we observe that

Let *γ*_*n*_=(1−*ρ*)*α*_*n*_ and . From condition (H3) and (H8), we have

By Lemma 5, we obtain

From (34), we have

It follows that

Let . For all , we get38

By Lemma 4, we have3940

and41

By Lemma 3(1), we obtain42

Now, from (38)-(42), it follows that43

since *v*_*n*_→0 and (*I*−*V*_*i*_)*y*_*n*_→0, as *n*→*∞*, then every weak cluster point of {*x*_*n*_} is also a strong cluster point. By Proposition 2, {*x*_*n*_} is bounded, thus there exists a subsequence  converging to *x*^∗^. For all  by (38), we compute44

Since *v*_*n*_→0,(*I*−*V*_*i*_)*y*_*n*_→0 for all *i*≥1, and ∥*u*_*n*_−*u*_*n*−1_∥/*α*_*n*_→0, letting *k*→*∞* in (44), we obtain

Since (49) has the unique solution, it follows that *ω*_*w*_(*x*_*n*_)={*x*^∗^}. Since every weak cluster point of {*x*_*n*_} is also a strong cluster point, we conclude that *x*_*n*_→*x*^∗^ as *n*→*∞*. This completes the proof.

If we take *T*_*i*_=*T*, for all *i*≥1, where *T*:*C*→*C* is a *k*-strict pseudo-contraction mapping in Theorem 1, then we get the following result: □

### Corollary 2

Let *C* be a nonempty closed and convex subset of a real Hilbert space *H*. Let *f*:*C*→*H* be a *ρ*-contraction mapping, *S*:*C*→*H* be a nonexpansive mapping and *T*:*C*→*C* be a *k*-strict pseudo-contraction mapping such that *F*(*T*)≠*∅*. Let *x*_1_ ∈ *C* and define the sequence {*x*_*n*_} by45

where *V*=*kI*+(1−*k*)*T*,{*α*_*n*_}⊂(0,1) and {*β*_*n*_}⊂(0,1) are sequences satisfying the conditions (H2) with *τ*=0, (H3), either (H4) and (H5), or (H6) and (H7). Then the sequence {*x*_*n*_} converges strongly to a point *z* ∈ *F*(*T*), which is the unique solution of the variational inequality:

Taking *k*_*i*_=0, for all *i*≥1 in Theorem 1, then we get the following result:

### Corollary 3

Let *C* be a nonempty closed and convex subset of a real Hilbert space *H*. Let *f*:*C*→*H* be a *ρ*-contraction mapping, *S*:*C*→*H* be a nonexpansive mapping and  be a countable family of nonexpansive mappings and . Let *α*_0_ = 1, *x*_1_ ∈ *C* and define the sequence {*x*_*n*_} by46

where {*α*_*n*_}⊂(0,1) and {*α*_*n*_} is a strictly decreasing sequence, {*β*_*n*_}⊂(0,1) and {*α*_*n*_} and {*β*_*n*_} are sequences satisfying the conditions (H2) with *τ*=0, (H3), either (H4) and (H5), or (H6) and (H7). Then the sequence {*x*_*n*_} converges strongly to a point , which is the unique solution of the variational inequality:

If we take *k*=0 in Corollary 2, then we get the following result:

### Corollary 4

Let *C* be a nonempty closed and convex subset of a real Hilbert space *H*. Let *f*:*C*→*H* be a *ρ*-contraction mapping, *S*:*C*→*H* be a nonexpansive mapping and *T*:*C*→*C* be a nonexpansive mapping such that *F*(*T*)≠*∅*. Let *x*_1_∈*C* and define the sequence {*x*_*n*_} by47

where {*α*_*n*_}⊂(0,1),{*β*_*n*_}⊂(0,1) and {*α*_*n*_} and {*β*_*n*_} are sequences satisfying the conditions (H2) with *τ*=0, (H3), either (H4) and (H5), or (H6) and (H7). Then the sequence {*x*_*n*_} converges strongly to a point *z* ∈ *F*(*T*), which is the unique solution of the variational inequality:

If we take *T*_*i*_=*T*, for all *i*≥1, where *T*:*C*→*C* is a *k*-strict pseudo-contraction mapping in Theorem 2, then we obtain the following result:

### Corollary 5

Let *C* be a nonempty closed and convex subset of a real Hilbert space *H*. Let *f*:*C*→*H* be a *ρ*-contraction mapping, *S*:*C*→*C* be a nonexpansive mapping and *T*:*C*→*C* be a *k*-strict pseudo-contraction mapping and . Let *x*_1_∈*C* and define the sequence {*x*_*n*_} by48

where *V*=*kI*+(1−*k*)*T*, {*α*_*n*_}⊂(0,1), {*β*_*n*_}⊂(0,1) and {*α*_*n*_} and {*β*_*n*_} are sequences satisfying the conditions (H2) with *τ* ∈ (0,*∞*), (H3), (H8) and (H9). Then the sequence {*x*_*n*_} converges strongly to a point , which is the unique solution of the variational inequality:49

If we take *k*_*i*_=0, for all *i*≥1 in Theorem 2, then we get the following result:

### Corollary 6

Let *C* be a nonempty closed and convex subset of a real Hilbert space *H*. Let *f*:*C*→*H* be a *ρ*-contraction mapping, *S*:*C*→*C* be a nonexpansive mapping and  be a countable family of nonexpansive mappings and . Let *α*_0_ = 1, *x*_1_ ∈ *C* and define the sequence {*x*_*n*_} by50

where {*α*_*n*_}⊂(0,1) and {*α*_*n*_} is a strictly decreasing sequence, {*β*_*n*_}⊂(0,1) and {*α*_*n*_} and {*β*_*n*_} are sequences satisfying the conditions (H2) with *τ* ∈ (0,*∞*), (H3), (H8) and (H9). Then the sequence {*x*_*n*_} converges strongly to a point , which is the unique solution of the variational inequality:51

If *k*=0 in Corollary 5, then we get the following Corollary:

### Corollary 7

Let *C* be a nonempty closed and convex subset of a real Hilbert space *H*. Let *f*:*C*→*H* be a *ρ*-contraction mapping, *S*,*T*:*C*→*C* be nonexpansive mappings and . Let *x*_1_∈*C* and define the sequence {*x*_*n*_} by52

where {*α*_*n*_}⊂(0,1), {*β*_*n*_}⊂(0,1) and {*α*_*n*_} and {*β*_*n*_} are sequences satisfying the conditions (H2) with *τ* ∈ (0,*∞*), (H3), (H8) and (H9). Then the sequence {*x*_*n*_} converges strongly to a point , which is the unique solution of the variational inequality:53

### Remark 2

Prototypes for the iterative parameters are, for example, *α*_*n*_=*n*^−*θ*^ and *β*_*n*_=*n*^−*ω*^ (with *θ*,*ω* > 0). Since |*α*_*n*_−*α*_*n*−1_|≈*n*^−*θ*^ and |*β*_*n*_−*β*_*n*−1_|≈*n*^−*ω*^, it is not difficult to prove that (*H*8) is satisfied for 0<*θ*,*ω*<1and (*H*9) is satisfied if *θ*+*ω*≤1.

### Remark 3

Theorem 1 and Theorem 2 extend and improve the result of Gu et al. (2011) from the countable family of nonexpansive mappings to more general the countable family of strictly pseudo contraction mappings.
